# The effect of *FOXA2 *rs1209523 on glucose-related phenotypes and risk of type 2 diabetes in Danish individuals

**DOI:** 10.1186/1471-2350-13-10

**Published:** 2012-02-12

**Authors:** Karina Banasik, Mette Hollensted, Ehm Andersson, Thomas Sparsø, Annelli Sandbæk, Torsten Lauritzen, Torben Jørgensen, Daniel R Witte, Oluf Pedersen, Torben Hansen

**Affiliations:** 1 The Novo Nordisk Foundation Center for Basic Metabolic Research, Section of Metabolic Genetics, Faculty of Health Sciences, University of Copenhagen, Copenhagen, Denmark; 2 Department of General Practice, University of Aarhus, Aarhus, Denmark; 3 Faculty of Health Sciences, University of Copenhagen, Copenhagen, Denmark; 4 Research Centre for Prevention and Health, Glostrup University Hospital, Glostrup, Denmark; 5 Steno Diabetes Center, Gentofte, Denmark; 6 Faculty of Health Sciences, University of Aarhus, Aarhus, Denmark; 7 Hagedorn Research Institute, Gentofte, Denmark; 8 Faculty of Health Sciences, University of Southern Denmark, Odense, Denmark

## Abstract

**Background:**

Variations within the *FOXA *family have been studied for a putative contribution to the risk of type 2 diabetes (T2D), and recently the minor T-allele of *FOXA2 *rs1209523 was reported to associate with decreased fasting plasma glucose levels in a study using a weighted false discovery rate control procedure to enhance the statistical power of genome wide association studies in detecting associations between low-frequency variants and a given trait.

Thus, the primary aim of this study was to investigate whether the minor T-allele of rs1205923 in *FOXA2 *associated with 1) decreased fasting plasma glucose and 2) a lower risk of developing T2D. Secondly, we investigated whether rs1205923 in *FOXA2 *associated with other glucose-related phenotypes.

**Methods:**

The variant was genotyped in Danish individuals from four different study populations using KASPar^® ^PCR SNP genotyping system. We examined for associations of the *FOXA2 *genotype with fasting plasma glucose and estimates of insulin release and insulin sensitivity following an oral glucose tolerance test in 6,162 Danish individuals from the population-based Inter99 study while association with T2D risk was assessed in 10,196 Danish individuals including four different study populations.

**Results:**

The *FOXA2 *rs1209523 was not associated with fasting plasma glucose (effect size (β) = -0.03 mmol/l (95%CI: -0.07; 0.01), *p *= 0.2) in glucose-tolerant individuals from the general Danish population. Furthermore, when employing a case-control setting the variant showed no association with T2D (odds ratio (OR) = 0.82 (95%CI: 0.62-1.07), *p *= 0.1) among Danish individuals. However, when we performed the analysis in a subset of 6,022 non-obese individuals (BMI < 30 kg/m^2^) an association with T2D was observed (OR = 0.68 (95%CI: 0.49-0.94), *p *= 0.02). Also, several indices of insulin release and β-cell function were associated with the minor T-allele of *FOXA2 *rs1209523 in non-obese individuals.

**Conclusions:**

We failed to replicate association of the minor T-allele of *FOXA2 *rs1209523 with fasting plasma glucose in a population based sample of glucose tolerant individuals. More extensive studies are needed in order to fully elucidate the potential role of *FOXA2 *in glucose homeostasis.

## Background

Type 2 diabetes (T2D) is a common and complex disease characterized by a state of hyperglycemia resulting from defects in insulin action combined with dysfunction of the pancreatic β-cell. Still, the underlying genetic factors affecting the susceptibility for developing T2D and associated complications remain poorly elucidated. The Forkhead box A2 gene (*FOXA2*) encodes an upstream activator of the β-cell transcription factor network and variation in this gene is hypothesized to play a role in T2D pathogenesis. Studies have shown that mice lacking *Foxa2 *in pancreatic β-cells are severely hypoglycaemic and show hypersecretion of insulin in response to both glucose and amino acid stimuli, suggesting that *Foxa2 *has to be present in pancreatic β-cells in order to sustain appropriate circulating levels of insulin as well as for maintaining glucose homeostasis [1].

In humans, variations within the *FOXA *family have been studied for a putative contribution to the risk of developing T2D. A study by Zhu *et al*. found no statistically significant association between *FOXA2 *variants and T2D in a Japanese study sample (*n *= 208) [2]. In a study by Navas and colleagues, all three *FOXA *genes were sequenced in a sample of 96 T2D patients with mixed ancestry; however, no mutations within the coding regions of any of the three genes were found, suggesting that mutations within *FOXA *genes are not a common cause of T2D [3]. A more recent study, however, reported an association between the *FOXA2 *rs1055080 and a reduced risk of T2D in a North Indian study sample (*n *= 1,656) [4]. In line with this, Xing *et al*. identified *FOXA2 *rs1209523 (in high linkage disequilibrium (LD) with rs1055080 (CEU; r^2 ^= 0.82)) employing a weighted false discovery rate control procedure to enhance the statistical power of genome wide association studies (GWAS) in detecting associations between low-frequency variants and fasting plasma glucose levels [5]. This low-frequency variant (rs1209523; minor T-allele frequency = 4.3%) was shown to be associated with lowered levels of fasting plasma glucose in both European Americans (*n *= 7,428, *p *= 1.3 × 10^-3^) and African Americans (*n *= 2,029, *p *= 6.7 × 10^-3^) [5]. A meta-analysis of the five included studies (*n *= 11,734) generated an estimated effect size of -1.31 mg/dl fasting plasma glucose level per minor allele (*p_all samples _* = 2.2 × 10^-11^) [5].

Therefore, the primary aim of the current study was to investigate whether the minor T-allele of rs1205923 in *FOXA2 *associated with 1) decreased fasting plasma glucose among 6,162 Danish individuals from the population-based Inter99 study, and 2) a lower risk of developing T2D in 10,196 Danish individuals including four different study populations. Secondly, we investigated whether rs1205923 in *FOXA2 *associated with other traits related to glucose metabolism that could explain the previously found associations with T2D-related metabolic traits.

## Methods

The study was conducted in accordance with the Helsinki Declaration and approved by the Ethical Committees of Copenhagen and Aarhus.

### Study participants

Participants were of Danish nationality and prior to participation, a written informed consent was obtained from all individuals. Participants examined in the present study were from four different study populations: 1) The Inter99 study is a population-based randomized non-pharmacological intervention study for the prevention of ischaemic heart disease from the Research Centre for Prevention and Health in Glostrup, Denmark (ClinicalTrials.gov NTC00289237) [6]. A total of 6,162 participants with available genotypes for rs1209523 were classified as having normal glucose tolerance (NGT) (*n = *4,567), impaired fasting glycemia (*n = *508), impaired glucose tolerance (*n *= 707), screen-detected T2D (*n *= 256), or previously diagnosed T2D (*n *= 124); 2) T2D patients recruited at Steno Diabetes Center (SDC) (*n *= 1,695); 3) A random sample of middle-aged glucose-tolerant participants examined at SDC (*n *= 730) and 4) T2D patients from the population-based, high-risk Addition Denmark screening and intervention study cohort (*n *= 1,609) (Anglo-Danish-Dutch Study of Intensive Treatment in People with Screen-Detected Diabetes in Primary Care) (ClinicalTrials.gov ID-no: NCT00237548). A standard 75 g oral glucose tolerance test (OGTT) was performed in participants from study group 1 and 3. T2D was diagnosed according to World Health Organization 1999 criteria.

Analyses of quantitative diabetes-related traits were performed in glucose-tolerant individuals (*n *= 4,567), as well as in non-obese (BMI < 30 kg/m^2^) individuals (*n *= 4,022) from study population 1. All T2D patients and glucose-tolerant individuals were included in the case-control study of T2D (*n *= 10,196). In the T2D case-control study of non-obese individuals, study participants with a BMI above 30 were excluded for both T2D individuals and glucose-tolerant controls.

### Genotyping

The rs1209523 of *FOXA2 *was genotyped using KASPar^® ^SNP Genotyping system (KBioscience, Hoddesdon, UK). The success rate was 97.3% with a 0.0% error rate estimated from re-genotyping of 972 replicate samples. The genotype distribution obeyed Hardy-Weinberg equilibrium in all study populations (*p *> 0.14).

#### Biochemical and anthropometric measures

Height and weight were measured in light indoor clothing and without shoes. Hip circumference was measured at its maximum, and waist circumference was measured in the upright position midway between the iliac crest and the lower costal margin [6] BMI was calculated as weight (kg)/height^2 ^(m^2^). Blood samples were drawn after a 12 h overnight fast. A glucose oxidase method was used to analyze plasma glucose (Granutest; Merck, Darmstadt, Germany). Serum insulin (excluding des-31,32 and intact proinsulin) was measured using the Autodelfia insulin kit (Perkin-Elmer/Wallac, Turku, Finland). Serum C-peptide concentrations were measured by a time-resolved fluoroimmunoassay (Auto-DELFIA C-peptide kit; Perkin-Elmer/Wallac, Turku, Finland). Homeostasis model assessment of insulin resistance (HOMA-IR) was calculated as: (fasting plasma glucose (mmol/l) × fasting serum insulin (pmol/l))/22.5, and homeostasis model assessment of β-cell function (HOMA-B) was calculated as: (20 × fasting serum insulin (pmol/l))/(fasting plasma glucose (mmol/l) - 3.5) [7]. Information on sex, BMI, plasma glucose levels, and serum insulin levels to time points 0, 30, and 120 min during an OGTT is used to calculate the BIGTT-acute insulin response (BIGTT-AIR) as well as the BIGTT-insulin sensitivity index (BIGTT-Si). These indices highly correlate with those obtained during an intravenous glucose tolerance test. The calculations were performed as previously described [8]. Insulinogenic index was calculated as: (serum insulin at 30 min (pmol/l) - fasting serum insulin (pmol/l))/plasma glucose at 30 min (mmol/l). Disposition index was calculated as insulinogenic index/HOMA-IR, and Matsuda whole body insulin sensitivity index (ISI_Matsuda_) was calculated as (10,000/√ (fasting plasma glucose × fasting serum insulin) × (mean plasma glucose × mean serum insulin during OGTT)) [9]. The trapezoidal method was used to estimate the area under the curve (AUC) for plasma glucose, serum insulin and serum C-peptide, and the AUC for insulin/AUC for glucose ratio was calculated as AUC for insulin/AUC for glucose.

### Statistical analysis

All statistical analyses were performed using R statistical software version 2.12.1 (available at http://www.rproject.org). A general linear model was applied to test quantitative traits in relation to genotype, using an additive genetic model and adjusting for age, sex, and BMI where appropriate. Prior to analyses, non-normally distributed data (measures of serum insulin, serum C-peptide levels, insulinogenic index, HOMA-IR, ISI_Matsuda_, AUC for insulin/AUC for glucose ratio, and BIGTT-AIR) were logarithmically transformed. Logistic regression was used to compare allele frequencies in the case-control analysis, and the analysis was adjusted for age, sex, and BMI. A *p*-value of less than 0.05 was considered statistically significant. The statistical power for detecting an effect on fasting plasma glucose of - 0.07205 mmol/l per allele corresponding to -1.31 mg/dl found by Xing and colleagues [5] was estimated using 1,000 simulations and a significance threshold of 0.05. Based on the allele frequency of the variant and the sample size of 4,368 non-diabetic successfully genotyped individuals, we estimated a statistical power of 93% to detect an association. For comparison, the statistical power to detect an effect on fasting plasma glucose of 0.06, 0.05, 0.04, or 0.03 mmol/l per allele were 79%, 66%, 46%, or 30%, respectively.

The statistical power calculation for the case-control analysis was done using CaTS, power calculations for large genetic association studies http://www.sph.umich.edu/csg/abecasis/cats/, and the statistical power to detect an OR of 0.85, 0.90, or 0.95 for rs1209523 of *FOXA2 *was estimated to be 50%, 26%, or 10%, respectively (significance level: *p *< 0.05, minor allele frequency (MAF) = 4%, estimated T2D prevalence in the background population = 0.08).

### Functional prediction

FASTSNP (Function Analysis and Selection Tool for Single Nucleotide Polymorphisms; http://fastsnp.ibms.sinica.edu.tw) was used to predict the function of rs1209523 in *FOXA2*.

### External data

Data on glycemic traits have been contributed by MAGIC investigators and have been downloaded from http://www.magicinvestigators.org[10]. Data on T2D were available through the DIAGRAM meta-analysis [11].

## Results and discussion

### Association with fasting plasma glucose

We analyzed the *FOXA2 *rs1209523 minor T-allele which is located in the promoter region of the gene and is predicted by FASTSNP to have a very low to medium functional effect on *FOXA2 *function, for association with fasting plasma glucose in glucose-tolerant individuals from the Inter99 study population. No statistically significant association was found (effect size (β) = -0.03 mmol/l (95%CI: -0.07; 0.01), *p *= 0.2) (Table 1). Considering the high statistical power to detect an association in the present study, this suggests that the variant may only have a minor effect on fasting plasma glucose levels among Danish individuals. Similarly, no associations were found between rs1209523 and plasma glucose levels after 30-min and 120-min during an OGTT (Table 1).

**Table 1:** Anthropometric and metabolic characteristics of successfully genotyped glucose-tolerant Danish individuals from the Inter99 study according to rs1209523 *FOXA2 *genotype

rs1209523	CC	CT	TT	β [95%CI]	*p*_add_
*n *(men/women)	4,091(1,880/2,211)	269(133/136)	8(6/2)		

Age (years) ^¥^	45.2 ± 7.9	44.8 ± 7.3	45.7 ± 7.2		

BMI^¥^	25.5 ± 4.1	25.5 ± 3.6	25.8 ± 2.4	-0.02 [-0.49; 0.45]	0.9

**Plasma glucose (mmol/l)**					

Fasting^¥^	5.3 ± 0.4	5.3 ± 0.4	5.6 ± 0.3	-0.03 [-0.07; 0.01]	0.2

30-min during an OGTT^¥^	8.2 ± 1.5	8.2 ± 1.5	8.9 ± 1.1	0.04 [-0.12; 0.21]	0.6

120-min during an OGTT^¥^	5.5 ± 1.1	5.5 ± 1.1	5.3 ± 1.2	-0.02 [-0.14;0.11]	0.8

Incremental AUC^¥^	181 ± 101	186 ± 98	185 ± 73	5.36 [-6.26; 16.98]	0.4

**Serum insulin (pmol/l)**					

Fasting	31 (22-45)	34 (23-49)	42 (30-50)	5.0% [-0.9; 10.8]	0.1

30-min during an OGTT	242 (175-344)	248 (188-346)	314 (232-425)	7.9% [2.1; 13.7]	0.008

120-min during an OGTT	136 (86-209)	137 (94-216)	212 (120-240)	9.3% [1.4; 17.3]	0.02

Incremental AUC	17,720 (12,540-25,020)	17,300 (13,680-25,800)	18,360 (14,250-28,120)	7.9% [1.6; 14.2]	0.01

**Serum C-peptide (pmol/l)**					

Fasting	493 (391-631)	520 (407-663)	602 (564-726)	3.8% [0.2; 7.5]	0.04

30-min during an OGTT	1,870 (1,470-2,360)	1,950 (1,580-2,412)	2,505 (1,685-2,738)	4.3% [0.4; 8.2]	0.03

120-min during an OGTT	1,960 (1,480-2,490)	1,990 (1,580-2,520)	2,485 (1,975-2,968)	5.0% [0.6; 9.3]	0.03

Incremental AUC	147,800 (117,200-183,200)	150,700 (122,700-185,400)	176,500 (150,000-199,400)	4.6% [0.7; 8.4]	0.02

**Derived indices**					

*β-cell function*					

Insulinogenic index	25.8 (17.9-37.6)	26.0 (19.6-39.0)	34.3 (19.7-42.4)	7.0% [0.5; 13.5]	0.03

BIGTT-AIR	1,672 (1,348-2,099)	1,710 (1,387-2,191)	1,455 (1,238-2,500)	3.5% [-1.1; 8.1]	0.1

HOMA-B	353 (248-508)	380 (267-560)	401 (310-488)	6.9% [0.9; 12.9]	0.02

AUCinsulin/AUCglucose	27.3 (20.2-37.4)	27.8 (21.1-39.7)	29.2 (23.2-40.8)	6.5% [1.2; 11.9]	0.02

*Insulin sensitivity*					

HOMA-IR	7.4 (5.2-10.9)	7.9 (5.4-11.8)	10.5 (7.8-11.8)	4.4% [-1.6; 10.4]	0.2

BIGTT-S_i_^¥^	10.4 ± 3.7	10.1 ± 3.7	8.6 ± 3.3	-0.33 [-0.78; 0.12]	0.2

ISI_Matsuda_	25.9 (18.2-35.6)	24.4 (16.8-34.5)	19.2 (15.0-26.5)	-5.8% [-11.2; -0.4]	0.03

*β-cell function corrected for insulin sensitivity*					

Disposition index	3.5 (2.3-5.2)	3.7 (2.4-5.4)	3.9 (3.0-4.2)	2.6% [-4.9; 10.1]	0.5

Data are unadjusted means ± SD^¥ ^or medians (interquartile range). Values of serum insulin, serum c-peptide, and insulin-derived indices were logarithmically transformed prior to statistical analyses, and their effect sizes are presented as the increase/decrease in percent. Effect sizes (β) and *p*-values shown are for an additive genetic model and are adjusted for age, sex, and BMI where appropriate. CI, confidence interval; add, additive; AUC, area under the curve.

The weighted false discovery rate control procedure applied by Xing and colleagues to enhance the statistical power of detecting associations with low frequency variants was shown to boost the *p*-values of variants with even small effect sizes to genome-wide significance [5]. As mentioned by the authors, the weighted false discovery rate control procedure is a prototype and its properties in GWAS should be further investigated. A large meta-analysis (*n *= 46,263) of European-ancestry-based GWAS on fasting glucose levels failed to identify a genome-wide significant association with *FOXA2 *(β = -0.028 mmol/l, *p *= 0.00394) [10]. This may, as Xing *et al*. suggest, be attributed to the low MAF of the variant which makes it less likely to detect an association compared to more frequent variants with a similar effect size. However, it is more likely that the effect size observed by Xing and colleagues is overestimated as an example of "winners curse", and a lower effect size for fasting plasma glucose would cause a considerably decreased power to detect associations in this study. Larger studies in study populations of different origin are needed to evaluate an effect of *FOXA2 *rs1209523 on fasting plasma glucose levels.

### Association with T2D

We investigated, in a case-control setting (*n*_cases_(CC/CT/TT): 3340/166/1 vs. *n*_controls_(CC/CT/TT): 4567/300/8), whether rs1209523 of *FOXA2 *associated with a decreased risk of developing T2D as indicated by the findings by Xing *et al*. [5]. No statistically significant association with T2D was found (odds ratio (OR) = 0.82 (95%CI: 0.62-1.07), *p *= 0.1) (Table 2). The OR, however, indicates that the minor T-allele of rs1209523 may exert a protective effect on T2D risk, although not statistically significant. Yet, the observed effect on T2D is not comparable to the one observed in the DIAGRAM meta-analysis (OR = 1.12 (0.94-1.34), *p *= 0.2) [11]. Considering the relatively low statistical power in our study, the indication of a protective effect of the variant on T2D is to be considered a chance-finding.

**Table 2:** Genotype distribution and allele frequency for *FOXA2 *rs1209523 among patients with T2D and glucose-tolerant control individuals

	*n (men/women)*	CC (%)	CT (%)	TT (%)	MAF (95% CI)	OR (95% CI)	*P*_add_
*All individuals*							

NGT	4,875 (2,256/2,619)	4,567 (93.7)	300 (6.2)	8 (0.2)	3.2 (2.9-3.6)	0.82 (0.62-1.07)	0.1

T2D	3,507 (2,084/1,423)	3,340 (95.2)	166 (4.7)	1 (0.1)	2.4 (2.1-2.8)		

*Non-obese individuals (BMI < 30 kg/m^2^)*							

NGT	4,292 (1,999/2,293)	4,012 (93.5)	272 (6.3)	8 (0.2)	3.4 (3.0-3.8)	0.68 (0.49-0.94)	0.02

T2D	1,730 (1,096/634)	1,653 (95.5)	77 (4.5)	0 (0)	2.2 (1.8-2.8)		

Number of individuals divided into genotype groups (% in each group), and frequencies of the minor allele (MAF) in percentages. Logistic regression was used to compare allele frequencies (*P_add_)*. The odds ratios (OR) and the 95% confidence interval (CI) are given for comparison of allele frequency. NGT: Glucose-tolerant individuals, T2D: type 2 diabetic patients.

On the contrary, our finding corresponded well to the findings by Tabassum and colleagues where a protective effect on T2D risk among normal-weight North Indians (*n *= 566) was observed for *FOXA2 *rs1055080 (OR = 0.59 (0.40-0.88), *p *= 0.011) [4]. Due to the strong LD between rs1055080 and rs1209523 (CEU; r^2 ^= 0.82) the association signals are likely to originate from the same causal variant. Therefore, we investigated whether rs1209523 of *FOXA2 *associated with a decreased risk of developing T2D among non-obese (BMI < 30 kg/m^2^) Danish individuals (*n*_cases(BMI < 30)_(CC/CT/TT): 1653/77/0 vs. *n*_controls(BMI < 30)_(CC/CT/TT): 4012/272/8) (Table 2). Here we found that rs1209523 of *FOXA2 *associated with a statistically significant decrease in the risk of developing T2D (OR = 0.68 (95%CI: 0.49-0.94), *p *= 0.02) (Table 2). Whether this is a true finding needs further investigation in other independent study populations.

### Associations with glucose-related phenotypes

When *FOXA2 *rs1209523 was analyzed for associations with glucose-related phenotypes, the T-allele significantly associated with increased serum insulin levels after 30-min (β = 7.9% (2.1; 13.7), *p *= 0.008) and 120-min (β = 9.3% (1.4; 17.3), *p *= 0.02) during an OGTT (Table 1). Furthermore, the rs1209523 T-allele associated with an increased incremental AUC for insulin (β = 7.9% (1.6; 14.2), *p *= 0.01) during the OGTT (Table 1). The associations were underpinned by significant associations for serum C-peptide levels during the OGTT (Table 1). In line with these results, several indices of insulin release and β-cell function showed significantly increased levels for the *FOXA2 *rs1209523 minor T-allele (Table 1). The indication of a general effect on β-cell function is supported by the findings reported by the MAGIC investigators where the T-allele of rs1209523 in *FOXA2 *significantly associated with an increased HOMA-B (β = 0.037 (standard error: 0.0093), *p *= 6.595 × 10^-5^).

To test whether these associations were independent of BMI, we conducted the analysis without adjusting for BMI, and the associations remained significant (data not shown). Likewise, stratifying the analysis according to BMI using a cut-off of 30 kg/m^2 ^showed that the significant associations were driven by the non-obese (BMI < 30 kg/m^2^) individuals (Table 3). However, these data should be interpreted with caution since no obese individuals were homozygous for the minor T-allele of rs1209523. Indices of insulin sensitivity (HOMA-IR and BIGTT-S_i_) showed no evidence of associations with *FOXA2 *rs1209523 among glucose-tolerant individuals.

**Table 3:** Anthropometric and metabolic characteristics of successfully genotyped non-obese (BMI < 30 kg/m^2^) glucose-tolerant Danish individuals from the Inter99 study according to rs1209523 *FOXA2 *genotype

rs1209523	CC	CT	TT	β [95%CI]	*p*_add_
*n *(men/women)	3,597(1,663/1,934)	244(121/123)	8(6/2)		

Age (years)^¥^	45.1 ± 7.9	44.7 ± 7.2	45.7 ± 7.2		

BMI^¥^	24.1 ± 7.9	24.7 ± 2.8	25.8 ± 2.4	0.32 [-0.004; 0.65]	0.05

**Plasma glucose (mmol/l)**					

Fasting^¥^	5.3 ± 0.4	5.2 ± 0.4	5.6 ± 0.3	-0.03 [-0.07; 0.02]	0.3

30-min during an OGTT^¥^	8.1 ± 1.5	8.2 ± 1.4	8.9 ± 1.1	0.05 [-0.13; 0.22]	0.6

120-min during an OGTT^¥^	5.4 ± 1.1	5.4 ± 1.1	5.3 ± 1.2	-0.01 [-0.15;0.12]	0.8

Incremental AUC^¥^	176 ± 102	182 ± 96	185 ± 73	5.64 [-6.64; 17.92]	0.4

**Serum insulin (pmol/l)**					

Fasting	29 (21-41)	32 (22-44)	42 (30-50)	6.5% [0.02; 13.0]	0.2

30-min during an OGTT	229 (168-321)	244 (186-327)	314 (232-425)	9.2% [2.9; 15.6]	0.01

120-min during an OGTT	131 (83-194)	135 (92-198)	212 (120-240)	10.9% [2.5; 19.4]	0.03

Incremental AUC	16,880 (12,140-23,300)	16,500 (13,240-23,430)	18,360 (14,250-28,120)	9.1% [2.4; 15.9]	0.02

**Serum C-peptide (pmol/l)**					

Fasting	473 (381-592)	495 (398-604)	602 (564-726)	4.7% [0.6; 8.8]	0.09

30-min during an OGTT	1,820 (1,440-2,280)	1,920 (1,525-2,315)	2,505 (1,685-2,738)	5.2% [1.0; 9.4]	0.05

120-min during an OGTT	1,900 (1,450-2,400)	1,950 (1,548-2,420)	2,485 (1,975-2,968)	5.8% [1.2; 10.4]	0.03

Incremental AUC	144,200 (115,100-177,800)	149,000 (122,000-182,000)	176,500 (150,000-199,400)	5.4% [1.3; 9.5]	0.03

**Derived indices**					

*β-cell function*					

Insulinogenic index	24.7 (17.3-35.7)	24.9 (18.5-36.9)	34.3 (19.7-42.4)	8.2% [1.2; 15.1]	0.04

BIGTT-AIR	1,596 (1,311-1,973)	1,665 (1,361-2,060)	1,455 (1,238-2,500)	5.7% [1.4; 10.0]	0.01

HOMA-B	337 (240-399)	367 (263-515)	401 (310-488)	6.7% [0.4; 13.1]	0.04

AUCinsulin/AUCglucose	26.0 (19.6-34.9)	27.0 (20.4-37.9)	29.2 (23.2-40.8)	7.7% [2.0; 13.5]	0.01

*Insulin sensitivity*					

HOMA-IR	7.0 (4.9-9.8)	7.5 (5.0-10.6)	10.5 (7.8-11.8)	5.9% [-0.8; 12.7]	0.2

BIGTT-S_i_^¥^	11.0 ± 3.3	10.6 ± 3.5	8.6 ± 3.3	-0.53 [-0.95; 0.10]	0.02

ISI_Matsuda_	27.4 (19.9-36.9)	26.6 (18.1-35.7)	19.2 (15.0-26.5)	-7.2% [-13.5; -1.1]	0.02

*β-cell function corrected for insulin sensitivity*					

Disposition index	3.6 (2.4-5.3)	3.9 (2.4-5.6)	3.9 (3.0-4.2)	2.5% [-5.4; 10.5]	0.5

Data are unadjusted means ± SD^¥ ^or medians (interquartile range). Values of serum insulin, serum c-peptide, and insulin-derived indices were logarithmically transformed prior to statistical analyses, and their effect sizes are presented as the increase/decrease in percent. Effect sizes (β) and *p*-values shown are for an additive genetic model and are adjusted for age and sex. CI, confidence interval; add, additive; AUC, area under the curve.

None of the identified associations in this study would remain significant after Bonferroni corrections.

Altogether, our findings may suggest that the improved β-cell function in carriers of the rs1209523 minor T-allele may play a role in the observed lowered fasting plasma glucose levels. A study by Gao *et al*. showed that inducible ablation of both *Foxa1 *and *Foxa2 *in mature mouse β-cells lead to impaired glucose homeostasis with impaired insulin secretion, suggesting that these transcription factors play a crucial role in the development and maintenance of β-cell specific secretory and metabolic pathways [12]. Interestingly, Gao *et al*. also showed that the gene encoding hydroxyacyl-coenzyme A dehydrogenase (*Hadh*) is down regulated 2.4-fold in *Foxa2 *deficient β-cells and 5.4-fold in β-cells lacking both *Foxa1 *and *Foxa2 *[12]. Decreased expression of the gene encoding a related mitochondrial fatty acid oxidation enzyme, the short-chain specific acyl-CoA dehydrogenase *(ACADS) *has been hypothesized to impair insulin secretion [13], and a study by our group has previously shown that the minor C-allele of rs2014355 in *ACADS *associated with reduced measures of glucose-stimulated insulin release [14]. Thus, altered *FOXA2 *expression may influence insulin secretion, potentially in combination with the regulation of genes expressed in the same molecular pathway.

## Conclusions

The *FOXA2 *rs1209523 was not significantly associated with fasting plasma glucose in glucose-tolerant individuals from the general Danish population of middle-aged people. Furthermore, when employing a case-control setting the T-allele was not found to be significantly associated with the risk of developing T2D. However, when we performed the analysis exclusively in non-obese individuals (BMI < 30 kg/m^2^), a significant association with T2D was observed. Also, several indices of insulin release and β-cell function showed significantly increased levels for the *FOXA2 *rs1209523 minor T-allele in the non-obese study population. More extensive studies are needed in order to fully elucidate the potential contribution of variation in *FOXA2 *to glucose homeostasis.

## List of abbreviations

AUC: Area under the curve; BIGTT-AIR: BIGTT-acute insulin response; BIGTT-Si: BIGTT-insulin sensitivity index; FOXA: Forkhead box A; GWAS: Genome-wide association study; HOMA-B: Homeostasis model assessment of β-cell function; HOMA-IR: Homeostasis model assessment of insulin resistance; IR: Insulin resistance; ISI_Matsuda_: Matsuda whole body insulin sensitivity index; LD: Linkage disequilibrium; OGTT: Oral glucose tolerance test; SDC: Steno Diabetes Center; SNP: Single nucleotide polymorphism; T2D: Type 2 diabetes.

## Competing interests

K. Banasik, T. Hansen and O. Pedersen hold employee shares in Novo Nordisk A/S. All other authors declare that there is no competing interest associated with this manuscript.

## Authors' contributions

The concept and idea regarding the epidemiological studies underlying the study populations was conceived by TJ, AS, TL, OP, and TH. The collection of study subjects was planned and performed by TJ, AS, TL, OP, and TH. The original hypothesis regarding the study was conceived by TH and approved by OP. Detailed planning of analyses and study design was performed by KB, MH and TS and approved by OP and TH. KB, MH, EA, DW, OP, and TH contributed to the establishment of study population databases specific for this study. Statistical analyses in association studies were performed by KB, MH, TS and EA. The first manuscript was written by KB and MH with equal contribution and the final draft was finalized by KB, MH, OP and TH. All authors revised the manuscript and contributed to the discussion. The final manuscript was read and approved by all authors.

## Pre-publication history

The pre-publication history for this paper can be accessed here:

http://www.biomedcentral.com/1471-2350/13/10/prepub
